# Prevalence and Risk Mapping of Intestinal Parasites in the “Hungry Valleys” Region of Slovakia

**DOI:** 10.3390/pathogens14100966

**Published:** 2025-09-24

**Authors:** Lukáš Ihnacik, Júlia Šmigová, Carmen Anthonj, Ingrid Papajová

**Affiliations:** 1 Institute of Parasitology, Slovak Academy of Sciences, Hlinkova 3, 040 01 Košice, Slovakia; ihnacik@saske.sk (L.I.); bystrianska@saske.sk (J.Š.); 2 Faculty of Geo-Information Science and Earth Observation (ITC), University of Twente, 7500 AE Enschede, The Netherlands; c.anthonj@utwente.nl

**Keywords:** helminthiases, marginalised communities, hotspot, health inequalities, risk mapping, Slovakia

## Abstract

Helminthiases remain a significant global health concern, affecting both the Global South and increasingly the Global North. In Slovakia, intestinal parasitic infections impact marginalised populations, particularly the population of Roma inhabitants, who often face inadequate housing, poor sanitation, and limited access to clean water. This study examines the prevalence of intestinal parasites in the “Hungry Valleys”, an economically challenged region of eastern Slovakia, with a higher number of Roma inhabitants. A total of 3816 stool samples were analysed using sedimentation methods, revealing an overall positivity rate of 5.06%. The highest prevalence was found among Roma inhabitants, with 23 times higher chance for infection than non-Roma inhabitants, rural residents, and children under 18. The most common parasites were *Ascaris lumbricoides* and Trichuris trichiura. Statistical analyses revealed strong associations between infection rates and factors such as density of the population of Roma inhabitants and inadequate access to water. Risk maps created in QGIS identified areas of high transmission. These findings highlight the urgent need for targeted public health interventions, especially in vulnerable Roma communities. Integrating spatial analysis with epidemiological data can guide more effective prevention efforts. Addressing structural inequalities is key to reducing the burden of parasitic diseases in marginalised populations.

## 1. Introduction

Endoparasites are parasites that live and reproduce within their hosts [1,2]. Among other species, these include helminths (parasitic worms) and intestinal protozoa. A subgroup that inhabits the intestines is referred to as intestinal parasites [3]. Notably, they are among the most common human parasites worldwide [4]. Their presence is closely linked to unhygienic living environments. They thrive in communities that lack basic amenities and sanitation services. Although global conditions related to drinking water, sanitation, and hygiene (WASH) have improved [5], parasitic infections and the diseases they cause continue to pose a persistent public health challenge, despite ongoing efforts to reduce exposure. They can cause serious intestinal parasitic diseases, often classified as neglected tropical diseases. These conditions are primarily associated with impoverished communities in tropical regions of the Global South. However, their distribution extends far beyond these regions, even reaching countries of the Global North in temperate zones, including Slovakia [6]. Although the overall prevalence of intestinal parasitoses in Slovakia is not alarmingly high, averaging below 15%, but some areas show considerably higher prevalence [7,8]. These areas are typically characterised by underdeveloped basic infrastructure and a higher concentration of marginalised groups. Among these, the population of Roma is the most represented in Slovakia. According to governmental studies and strategic documents [9], the population of Roma in Slovakia faces significant social and economic challenges, including poverty, exclusion, and discrimination. Many members of the Roma communities are, due to various factors, excluded from the majority of villages and pushed to the outskirts, where they are living in concentrated settlements [10]. Living conditions, especially in the most isolated settlements, are often substandard [9,11]. They are characterised by a higher concentration of people (averaging 8 people per dwelling) living in inadequate housing [11]. Overcrowding is not the only challenge in Roma settlements, as poor infrastructure is also common. According to the summary in the Atlas of Roma Communities 2019 (from now on it will be referred to in the text only as “the Atlas”) [11], nearly 64% of inhabitants in Roma settlements use public water supply, while only 40% have access to sewerage systems [10]. Overall, this social exclusion puts communities closer to the forests or fields. This exposes people to more frequent contact with wild animals, rodents, stray dogs and cats, which could potentially also be a source of various parasitic diseases [12,13]. On top of that, people share their living conditions with pets (dogs, cats) [14]. These animals, without proper veterinary care or regular deworming, could also become sources of environmental contamination [12]. The above-mentioned facts significantly increase the risk of transmission of both infectious and parasitic diseases from animals to humans in affected areas [15]. The most effective protection is prevention through awareness of infection pathways. However, this requires a clear understanding of the epidemiological situation (what), the risk areas (where, and the main risk factors (why). Such information allows for targeted interventions to reduce transmission (how). Therefore, this study analyses the epidemiological situation in an area of “Hungry Valleys” using parasitological methods. Additionally, correlation and spatial analysis are used to identify risk factors and determine areas with the highest risk of transmission and spread of intestinal parasitosis.

## 2. Materials and Methods

### 2.1. Study Area

**Figure 1:**
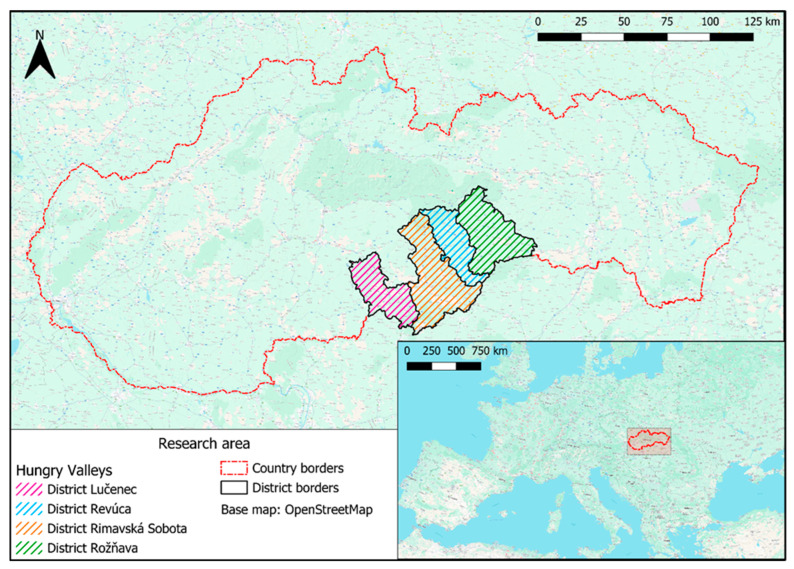
Location of our research area of “Hungry Valleys” in Slovakia and location of Slovakia in Europe. Created by Lukáš Ihnacik with the use of QGIS 3.40 LTR Bratislava.

In this research, we focused on a specific region of Slovakia, namely the districts of Lučenec, Rožňava, Rimavská Sobota, and Revúca (Figure 1). These districts consist of a total of 258 villages and 10 towns. They are among several districts often colloquially referred to as the “Hungry Valleys” due to their numerous social and economic challenges [16]. In the past, these regions were important and thriving mining hubs. However, this industry has declined over the years and, in most parts, has completely disappeared. As a result, these regions struggle economically, with underdeveloped infrastructure and a shortage of job opportunities. This leads to consistently high unemployment rates, exceeding 15% for several years, nearly three times the nationwide average (around 6%), according to data from the Office of Labour, Social Affairs and Family in Slovakia [17]. Apart from that, these districts have one of the highest numbers of people with Roma ethnicity in all of Slovakia, where the population of Roma inhabitants makes up between 10% and 30% of the total population, as stated by the Atlas [11]. All of this contributes to several other problems connected with limited job opportunities, inadequate infrastructure, lower access to quality education and healthcare, and a lack of investment in local development. These aforementioned problems could potentially contribute to a higher incidence of diseases.

### 2.2. Data Collection

#### Primary Data Collection

A total of 3816 human stool samples were collected from inhabitants of 269 places in this region. We carried out the collection process from September 2021 to August 2024. Samples were obtained either directly from volunteers or through collaboration with local paediatricians or general practitioners. This approach enabled the collection of a large number of samples and ensured representation from both asymptomatic individuals and symptomatic patients. With such a collection, we tried to minimise potential sampling bias. Each sample was accompanied by a signed informed consent form containing basic demographic information about the volunteer, including age, gender, address, and self-identified minority or majority residency status. In the case of child participants, informed consent was obtained from the legal guardian, who also authorised the child’s participation in the research. Such information was essential for including their sample in the study. Samples lacking a signature or missing more than one of these criteria were excluded from the analysis. Additionally, all sensitive data was stored locally on drives and carefully managed. They were accessible only to one authorised personnel from the research team, and all procedures were conducted in compliance with GDPR and relevant ethical guidelines. Also, data were not shared online, using email or cloud storage, but rather used on one computer. With this approach, we minimised the chance of sensitive data leaks. Special attention was given to data governance, privacy, and ethical considerations throughout the study. All collected information was aggregated and anonymised at the village level to ensure that no individuals could be identified or recognised from the published data. When handling sensitive variables such as gender, age, ethnicity, and place of residence, only generalised counts were analysed. Personal identifiers were removed, and small-area data were excluded to prevent deductive disclosure. Informed consent forms were stored securely for five years, allowing participants the option to withdraw at any time. These measures ensured that both privacy and data integrity were maintained, while enabling meaningful analysis of public health risks at the community level. The entire research process (data collection, handling, and analysis) was approved by the Ethics Committee of the Košice Self-governing Region (document no. 5436/2019/ODDZ-25820, approved on 24 September 2019).

### 2.3. Secondary Data Collection

Comprehensive information on the population of Roma inhabitants was sourced from the Atlas [11]. This publication is one of its kind and provides detailed insights into the living, hygiene and housing conditions of marginalised communities across Slovakia. It is important to note that not all of Slovakia’s villages are included, as the data only considers municipalities with at least 30 Roma inhabitants or where Roma make up 30% of the population. Nevertheless, this publication provides valuable information necessary for risk map creation. Other demographic information and data about villages, categorised at the village boundaries level (LAU 2/administrative level 6), were sourced from the 2021 nationwide census conducted in Slovakia [18]. Additional geographic data, including settlement locations with exact coordinates, were obtained from the Czech mapping portal, Mapy.cz. A total of 9 distinct factors were selected from multiple sources for risk factor analysis. These included variables such as calculated overall population density, Roma population density, next settlement locations, access to drinking water, use of sewage systems and use of sumps in villages and settlements.

### 2.4. Data Analysis

#### Parasitological Analysis

All samples were immediately transported for parasitological examination to the laboratory at the Institute of Parasitology of the Slovak Academy of Sciences in Košice, where they were stored without preservation at 4 °C. Parasitological analyses were performed within 24–48 h. Stool samples were coprologically analysed using two sedimentation methods due to their different sensitivity. Most samples were analysed by using a modified concentration method with a SAF (sodium acetate, acetic acid, and formalin) solution for the detection of helminth eggs and protozoan cysts. Briefly, approximately 1–2 g of faeces were suspended in SAF solution, strained through medical gauze into a centrifuge tube, and centrifuged at 500× *g* for 1 min (Eppendorf 5408, Eppendorf SE, Hamburg, Germany). The supernatant was discarded, and the sediment was mixed with 7 mL of 0.85% NaCl and 2–3 mL of diethyl ether, shaken vigorously, and centrifuged again at 500× *g* for 5 min. After discarding the top layers, the sediment was examined microscopically (Leica DM 5000B, Leica Microsystems, Wetzlar, Germany) at 100× and 400× for helminths, and at 1000× with immersion oil for protozoa. Some samples were also analysed using the commercially available sedimentation kit Paraprep L (DiaMondiaL EEIG, Vienna, Austria). We followed the manufacturer’s instructions for this analysis. Briefly, ~0.5 g of stool was mixed with 2 mL of ethyl acetate and 6 mL of 10% formalin in a mixing tube connected to a collection tube with a filter. After 24 h at room temperature, samples were centrifuged at 500× *g* for 1 min. The supernatant was discarded, and the sediment was examined microscopically at 100× and 400× for the presence of helminth eggs.

### 2.5. Statistical Analysis

All data obtained from the census, the Atlas, informed consent forms, and parasitological analyses were organised into MS Excel 2408 (Microsoft Office LTSC Professional Plus 2024, Redmond, DC, USA) spreadsheets. The data were categorised using basic filtering and sorting functions. Initially, the samples were categorised by ethnicity into two groups (Roma ethnicity and non-Roma). Subsequently, the data were categorised by gender (men and women) and by the type of environment in which the participants lived (urban areas, such as towns/cities, and rural areas, such as villages). This division allowed for the observation of differences in positivity rates across various demographic and environmental factors and living environments. Firstly, key epidemiological metrics were calculated using RStudio 2024.12,1+563 (Posit PBC, Boston, MA, USA), including positivity rates, confidence intervals, and crude odds ratios. Lastly, *p*-values and Chi-squared values calculated from the Chi-squared test of independence were used to assess the statistical significance of these associations, allowing us to reject the hypothesis that observed differences occurred by random chance. Additionally, the dataset was uploaded into IBM SPSS Statistics 29.0.2.0 (IBM, Armonk, NY, USA), where the importance of the selected factors was determined using Spearman’s correlation analysis function, comparing positivity rates in individual villages with the data of selected factors. The resulting correlation coefficients were then converted to a percentage value, where the threshold value of 10% of the correlation coefficient share was determined (based on analysis in our previous research [8]). All factors that exceeded this value were considered important and they have an impact on the number of positive samples, whether increasing or decreasing the positivity rate. This analysis also provided us with the basis for the weighting of factors in later stages.

### 2.6. Spatial Analysis

The modified databases containing parasitological and demographic data were converted to comma-separated values (CSV) format. Subsequently, along with other necessary layers, they were uploaded into QGIS 3.40 LTR Bratislava (QGIS Development Team) software for analysis. All maps were created using a uniform coordinate reference system (CRS), specifically EPSG:5513—S-JTSK/Krovak. A map of positivity rates for each village was created using point data, where points depicted the centre of villages. As a point-based positivity map does not account for the free movement of residents, an interpolation of the points data was conducted to generate a comprehensive surface representation of positivity across the region. This approach ensures positivity is represented as a gradient across neighbouring areas, offering a more accurate spatial depiction. Later databases were converted to the UTM coordinate system for a specific part of Europe (UTM zone 34N) and imported into GeoDA 1.22 (GeoDa Center for Geospatial Analysis and Computatio; The University of Chicago, USA) software. This software was utilised to identify clustering patterns through the Spatial Autocorrelation function (Global Moran’s I). Through spatial autocorrelation analysis, we evaluated whether our high or low values in the data are spatially grouped, dispersed, or randomly distributed. Before creating the final risk map in QGIS, the data values for the selected factors were reclassified onto a common scale from 1 to 5, where 1 represented the best living conditions (e.g., higher utility usage or lower population density) and 5 indicated the worst living conditions. Intermediate values reflected gradations between these extremes. This reclassification was performed using the Reclassify tool, applying natural breaks (Jenks) to determine category boundaries and divide the data into five meaningful groups. Weighted and unweighted risk maps were then created by stacking the reclassified factor layers and combining their values using the raster calculator function in QGIS. In the raster calculator, unweighted maps were generated using the expression factor_1_ + factor_2_ + … + factor_n_. For weighted maps, the value of each factor was multiplied by its corresponding weight, using the expression weight_1_ × factor_1_ + weight_2_ × factor_2_ + … + weight_n_ × factor_n_. This comprehensive analysis enabled us to better understand the distribution of intestinal parasites across the population and identify critical risk factors contributing to the spread of infections, as well as high-risk areas where transmission is theoretically the most likely.

## 3. Results

Overall, 3816 human stool samples were analysed for the presence of the parasitic propagative stage. They were detected in 193 samples, accounting for 5.06% of the total number of human stool samples. The most prevalent were the eggs of *Ascaris lumbricoides*. Less common were eggs of *Trichuris trichiura* and *Enterobius vermicularis* (Table 1). When the samples were divided based on ethnicity, a significantly higher prevalence of samples with parasitic propagative stages were observed among individuals from the Roma ethnicity. More than 13% of the 1302 samples collected from the population of Roma individuals tested positive for endoparasitic infections, compared to just 0.68% of the 2514 samples from the majority population (Table 2). This represents a 22.95-fold higher likelihood of infection among the population of Roma compared to the majority population. Similarly, samples were divided by environment into samples from towns and samples from villages (Table 2). Significantly higher prevalence of positive cases was observed in samples from rural areas, where nearly 7% of samples collected from villages were positive for the presence of propagative stages of parasites, compared to only 3.46% of samples from urban areas (Table 2). This indicates that the likelihood of endoparasite infection is twice as high in rural areas than in urban areas. In contrast, when samples were divided by gender, no significant difference was observed, as both genders exhibited a similar positivity rate of approximately 5% (Table 2).

Lastly, the samples were divided according to the age structure into 5 different groups: infants (0–1 years), kids (2–6 years), adolescents (7–18 years), productive age (19–66), and post-productive age (over 66 years). The highest positivity was observed in adolescents, followed by infants and kids. In contrast, the productive and post-productive age groups have just a 2.01% and 0.74% positivity rate, respectively (Table 3).

**Figure 2:**
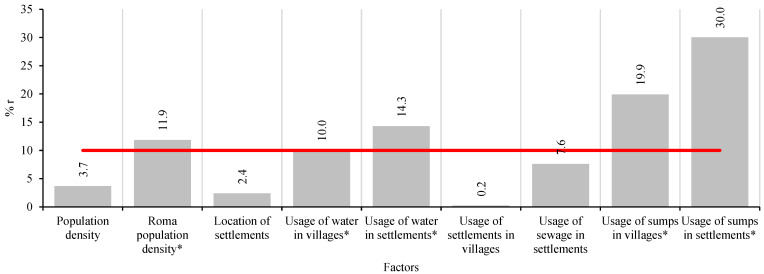
Percentage representation of Correlation coefficients (% r) of selected factors. The red line is the target line set at 10% of the share of the correlation coefficient. *—factors above 10% of correlation share.

All of the 9 factors (population density, Roma population density, location of settlements, access to water in villages and settlements, usage of sewage in villages and settlements and usage of sumps in villages and settlements in different villages of this region) were included in Spearman’s correlation analysis. The recalculated values are presented in Figure 2, which illustrates the share of influence of the correlation coefficient on overall positivity. As shown in Figure 2, the Roma population density, usage of water in villages and settlements, and usage of sumps in villages and settlements are above our threshold line of 10% of correlation coefficient share. While the density of population of Roma individuals and access to water in villages were exactly or slightly above the threshold, the usage of water in settlements and usage of sumps in villages and settlements showed higher shares, indicating their strong influence on parasitic prevalence in the region.

**Figure 3:**
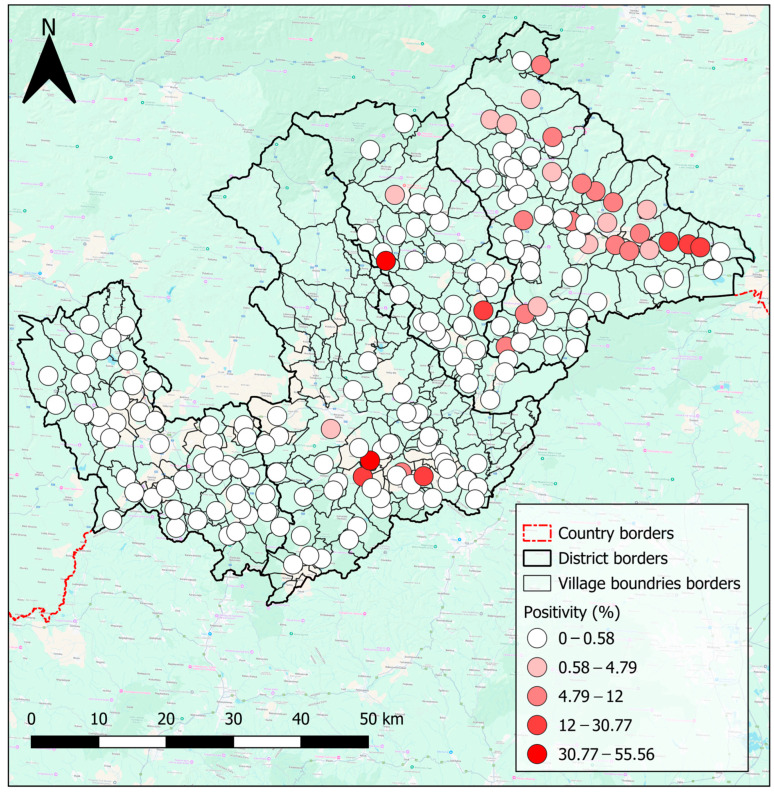
Map of the percentage of positive samples in the districts of “Hungry Valleys”. Each point depicting centre of the villages and different shades of red depict different percentage values. Created by Lukáš Ihnacik with the use of QGIS 3.40 LTR Bratislava.

The map of endoparasite positivity by villages (Figure 3) was created with the use of natural breaks as a way to categorise values in the legend, and a suitable colour palette was chosen to enhance visual clarity. Results reveal that the highest positivity rates were mostly towards the eastern part of the study area. Most notably in the Rožňava region, with additional hotspots in the southern Rimavská Sobota region and central Revúca region (Figure 3). Isolated islands with elevated positivity rates were also scattered across the Rožňava, Revúca, and Rimavská Sobota regions (Figure 3). Notably, all samples from the Lučenec region were analysed as negative. However, it is important to highlight that in the Rimavská Sobota region, samples were collected from only half of the villages, and some areas in these four regions had relatively low sample sizes. These limitations may influence the accuracy and representativeness of the results.

**Figure 4:**
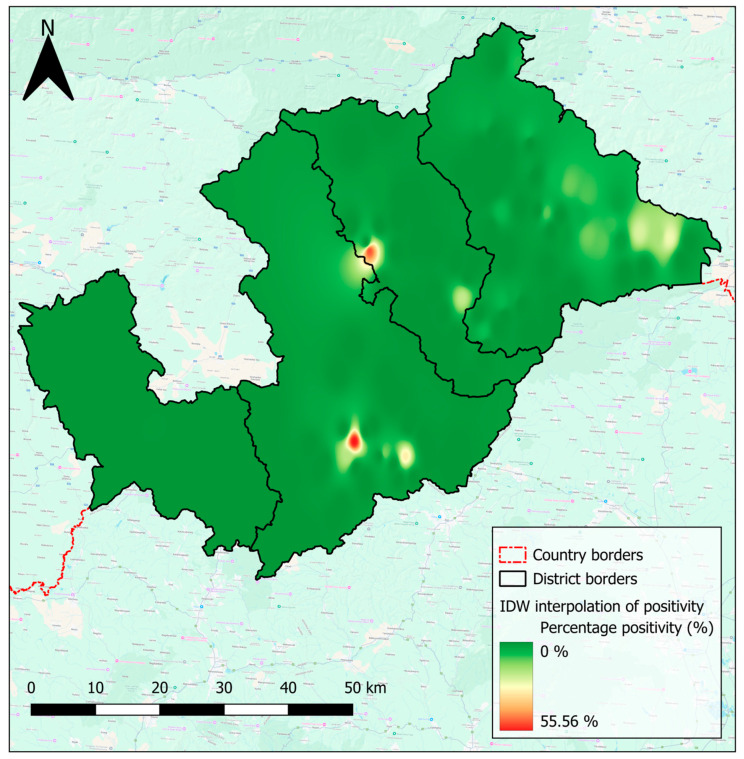
Interpolated map of percentage of positive samples in districts of “Hungry Valleys”. Created by Lukáš Ihnacik with use of QGIS 3.40 LTR Bratislava.

The created interpolated positivity map for each village better highlights distinct patterns of endoparasitic infection across the region (Figure 4). The use of a colour palette is highly effective, with shades of green representing lower positivity, shades of yellow and orange indicating moderate positivity, and shades of red highlighting high positivity. This gradient provides a clear and intuitive visualisation of areas with varying risk levels, making it easier to identify regions with higher or lower positivity rates. Higher positivity rates were observable in the eastern part of the Rožňava region, represented by bright green and yellow colours (Figure 4). Extremely high positivity rates were displayed as bright red and are observable in parts of the Rimavská Sobota and Revúca regions (Figure 4). Additionally, there are isolated yellow and orange areas indicating regions with moderately high positivity rates. Conversely, a lower to no number of positive samples, shown as a dark green colour, was recorded mainly in the western part of the area (Figure 4).

**Figure 5:**
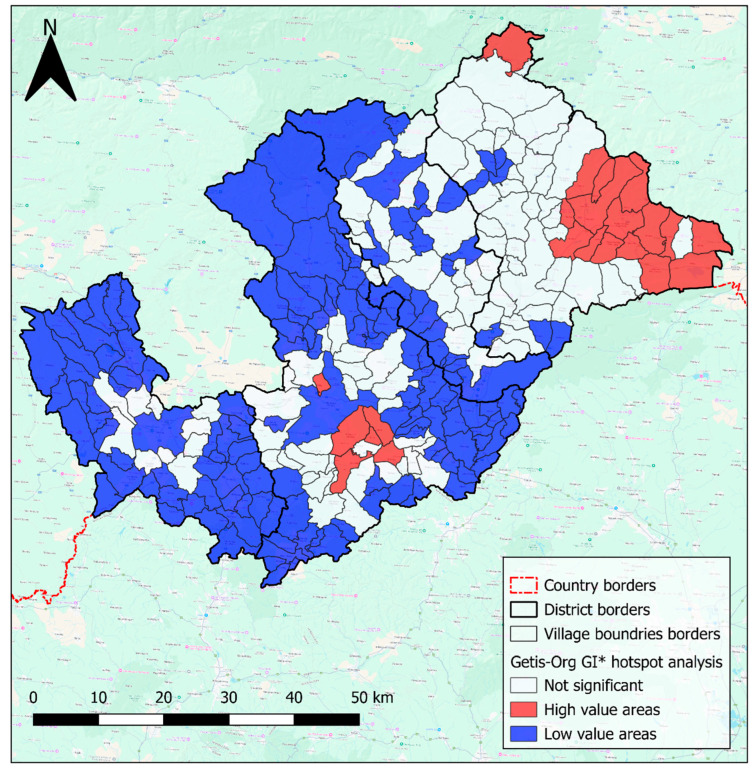
The results of Hot Spot Analysis (Getis-Ord Gi*). Created by Lukáš Ihnacik with the use of GeoDa 1.22 and QGIS 3.40 LTR Bratislava.

The results of the Hot Spot Analysis (Getis-Ord Gi*) indicate that points with high and low values are spatially clustered rather than randomly distributed. To enhance the visualisation of areas with clusters of high or low positivity values, the results from GeoDA 1.22 software were imported into QGIS, which offers greater flexibility for adjustments. As shown in Figure 5, significant hotspots are concentrated in the southeastern part of the study area, with a particularly prominent hotspot in the southern Rimavská Sobota region, represented in red. In contrast, cold spots, represented in blue, are primarily located in the western part of the study area, often in regions with a sparse number of samples (Figure 5).

**Figure 6:**
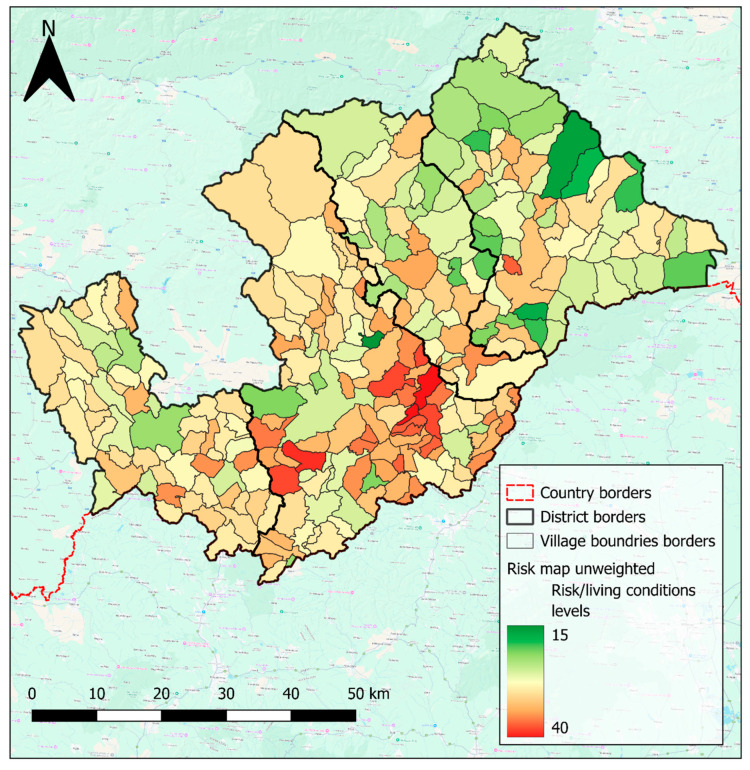
Unweighted risk map. The scale shows the values of the resulting unweighted risk (lower values are better). Created by Lukáš Ihnacik with use of QGIS 3.40 LTR Bratislava.

The unweighted risk map, created by summing the reclassified values of selected factors, could also be interpreted as an indicator of living conditions. These reclassified values represent factors such as access to utilities and population density, where lower overall values suggest better overall living conditions and higher overall values reflect poorer conditions. The final unweighted risk map (Figure 6) reveals that the highest values, indicative of the worst living conditions, are predominantly concentrated in the southern part of the Rimavská Sobota region, represented by darker shades of orange and red (Figure 6). The western part of the study area also shows elevated risk levels, indicating poorer living conditions, visible as lighter orange and yellow shades (Figure 6). In contrast, the eastern part of the study area demonstrates lower unweighted risk values, corresponding to better living conditions, represented by lighter green shades (Figure 6).

**Figure 7:**
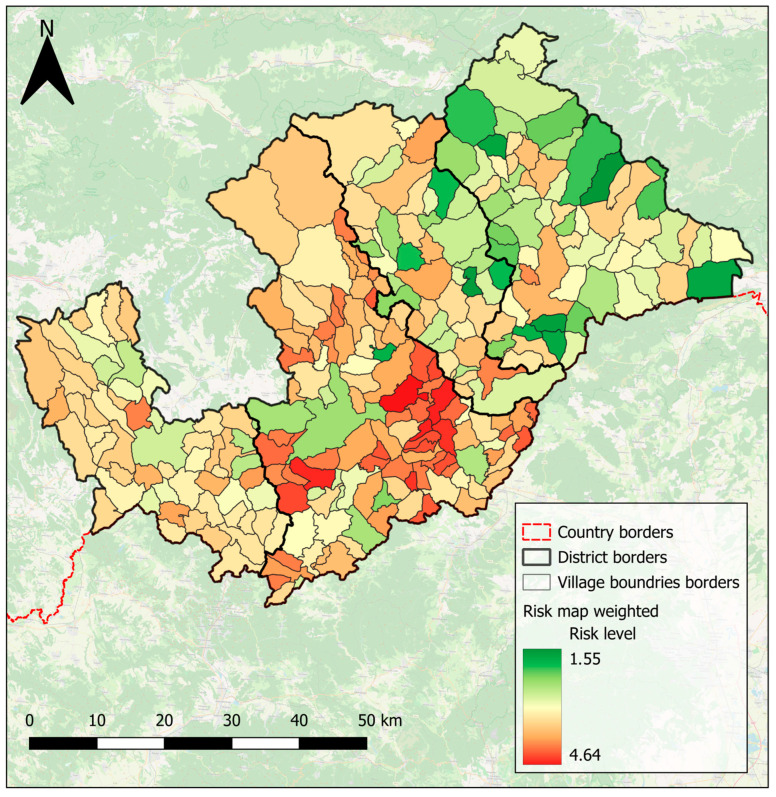
Weighted risk map. The scale shows the values of the resulting weighted risk (lower values are better). Created by Lukáš Ihnacik with use of QGIS 3.40 LTR Bratislava.

Similarly to the unweighted maps, weighted maps were generated, with the difference that weights were assigned to each factor before combining them. As shown in Figure 7, the study area can be distinctly divided into two parts. The western part exhibits a higher overall weighted risk of transmission and spread of endoparasitoses, represented on the map with shades of red and darker orange (Figure 7). In contrast, the eastern part shows a lower overall weighted risk, indicated by shades of green and yellow (Figure 7). Notably, isolated areas of both high and low risk are visible within the western and eastern parts of the district, highlighting localised variations in risk levels (Figure 7).

## 4. Discussion

### 4.1. Prevalence of Intestinal Parasites

It was confirmed that the overall prevalence of intestinal endoparasites in the human population in the studied region is relatively low, with the finding of intestinal parasite propagation stages in just over 5% of the total number of human stool samples. The most prevalent parasites were *A. lumbricoides*, present in 83% of positive samples, and *T. trichiura*, present in 11% of positive samples. The least detected were eggs of *E. vermicularis*, found in 7.25% of positive samples. These findings are similar to those reported in the study of Papajová et al. [19]. They also reported that eggs of A. lumbricoides and T. trichiura were the most prevalent, and they were detected in 70% and 6% of all positive cases, respectively. The least abundant were eggs of *E. vermicularis*, found in only 4% of positive samples. A key difference was that this research was carried out only in 32 different localities in eastern Slovakia. This observed similarity is likely due to the use of the same detection methods as in our study (Paraprep L kit and SAF method). Also, Dudlová et al. [20] confirmed in their study that *A. lumbricoides* and *T. trichiura* eggs were the most prevalent, found in 55% and 12% of positive samples, respectively. They also used the Paraprep L sedimentation kit and an unmodified ether-formalin sedimentation method for detection. In addition, unlike our study, their research also focused on detecting protozoan cysts and oocysts. Lastly, the research area was different and focused on several selected sites across Slovakia. Even though we did not perform perineal swabs or plastic tape tests, which are the standard Enterobius detection methods [21,22], we still found the presence of E. vermicularis eggs in almost 4% of all positive samples. Detection of *E. vermicularis* eggs by traditional concentration methods is uncommon and seldom occurs, as the adult female parasite generally lays eggs on the skin surrounding the anal opening rather than directly into the lumen of the intestine. This atypical finding suggests that these cases in question may have been severe infections in which large numbers of eggs were passed into the faeces during defecation.

### 4.2. Risk Varies According to the Sub-Population

When analysing human stool samples by ethnicity, we detected a significantly higher prevalence of positive cases among the population of Roma individuals (13.50%) compared to the majority population (0.76%). This was expected, as the living conditions and the hygiene levels are generally lower in segregated settlements than in the main part of the municipality [8,9,10,15]. Similarly, Štrkolcová et al. [23] found a higher prevalence of intestinal parasites in samples from the Roma infants (12.71%). In samples from the majority population, no positive cases were detected. Štrkolcová et al. [24] conducted another study on children and adolescents in the village of Medzev. This study also confirmed a higher prevalence of parasites among the population of Roma individuals. They found parasites in 80% of samples from Roma children living in a segregated settlement and in 70% of samples from an orphanage. On the other hand, parasites were confirmed in only 3.25% of samples from children living outside the settlement. Although these studies focused on children and infants, they confirm that poorer hygiene standards are in segregated settlements. Numerous studies in Slovakia support these findings, highlighting the high prevalence of endoparasites in marginalised communities [7,8,25,26]. When we evaluated the samples by sex, we did not observe a significant difference in parasite prevalence between the sexes. Both sexes had approximately the same number of positive samples, accounting for around 5% positivity. This result was consistent with the study of Pipiková et al. [26], who also found no significant difference between the sexes of children. In addition, by analysing human stool samples based on living environment, we found significantly more positive samples in a rural area (6.82%) compared to urban areas (3.46%). Likewise, Pipiková et al. [26] reported a higher prevalence of parasitic infection in children living in rural areas (28.36%) than those from urban areas (10.53%). Similarly, Ihnacik et al. [8] observed a higher prevalence of parasites in samples of residents living in a rural environment (33%) than in those living in an urban environment (18%). With age groups, we observed a higher number of positive samples in individuals under 18 years old than in those over 18. This outcome was expected, as younger individuals often gather in large groups and have not yet developed consistent hygiene practices. Dudlova et al. [20] also confirmed that the prevalence of helminths as well as protozoans was higher in individuals under 18 years of age. The helminth infections were less common in individuals over 18.

### 4.3. Determinants of Intestinal Parasite Transmission Risk

We used a simple correlation coefficient method to determine risk factors for the spread and transmission of intestinal parasites in the “Hungry Valleys” districts. The disadvantage was that it required information on the epidemiological situation in the study area. The most important factors identified by this method were the density of the Roma population, water usage and septic tank usage. Pullan et al. [27], in their analysis of risk factors for the spread of human helminths in a Brazilian community, identified socioeconomic status, toilet facilities (biological waste liquidation) and household crowding as significant contributors responsible for helminth infection. Their study employed a more advanced Bayesian Monte Carlo Markov Chain approach to model these associations. Similarly, Ross et al. [28], in their research on identifying risk factors for transmission of human helminthiasis in rural Philippines, found that low socio-economic status, poor sanitation, and proximity to water sources were the most influential factors. Additionally, low levels of education, occupation (i.e., farming and fishing), and male sex were all reliable indirect risk factors. Similarly, the Chi-square test was used in our study to explore associations between demographic and socio-economic characteristics and the likelihood of having any STH infection. In Slovakia, our earlier efforts to identify risk factors are published in the study by Ihnacik et al. [8]. Here we also employed correlation coefficients to analyse associations. However, in this study, our focus was expanded beyond risk factors for parasite transmission and spread in the human population to also assess risk factors in the dog population and the environment of selected locations in eastern Slovakia. Similarly, Ihnacik et al. [8] and Anthonj et al. [15] highlighted the density of the population of Roma individuals and access to water supply as important contributing factors. From this, we can say that factors related to water use and biological waste disposal are considered important and have an impact on the occurrence of intestinal parasites. In this case, also belonging to the Roma ethnic group influences the spread of parasites in Slovakia, as it affects the socio-economic status of the population. These findings suggest that these factors are important and likely contribute to the higher positivity rates. However, while they indicate a strong association with positivity rates, they do not demonstrate causality, only the strength and direction of the relationship. Also, it is worth noting that in this study, meteorological variables or environment information are less important, as Slovakia is located in a relatively uniform climate and environmental zone. Regional differences are not significant enough to fundamentally affect the occurrence of endoparasites and therefore were omitted from this analysis.

### 4.4. Risk Maps Highlight High-Risk Areas, and Inform Decision-Making in the Hungry Valleys

By risk mapping, we were able to identify the areas of the “Hungry Valleys” that are at the highest risk of parasite transmission and spread. The resulting risk maps highlight these high-risk areas, providing practical information to local authorities and mayors. In both the weighted and unweighted risk maps, the highest risk was notably concentrated in the south-central part of this area. They are outlining not only the most vulnerable areas to parasite transmission but are also characterised by the poorest living conditions. Earlier research by Papajová et al. [29] and Blišťan et al. [30] pioneered risk mapping in Slovakia by comparing different methods for weighting risk factors to develop risk maps for the transmission and spread of parasites in the human population. Their study incorporated variables such as water access, sanitation and hygiene conditions, educational level, population density, and age distribution. Lin and Wen [31] further explored the application of various spatial methods, emphasising that these approaches can offer deeper insights beyond those provided by traditional epidemiological analyses.

### 4.5. Implications of This Research for Other Marginalized Population Groups in Challenging Living Environments

Although this study focused on Roma communities in eastern Slovakia, the findings have broader relevance for other marginalized populations living in disadvantaged living environments (e.g., homelessness people, refugees camp). The strong associations observed between parasitic infection prevalence and factors such as inadequate sanitation, poor housing, and limited access to clean water mirror patterns are seen in communities globally (from informal settlements in high-income countries to rural, low-resource areas in the Global South). Our integrative approach, combining epidemiological, demographic, and spatial data, offers a method for identifying risk areas and risk factors for transmission and spread of endoparasitic diseases. It can be used, for example, in refugee camps or migrant settlements, which often experience environmental risk factors, limited public infrastructure, and systemic neglect. Beyond academic relevance, these insights also carry practical implications for policymakers and public health authorities. Ensuring access to safe water and sanitation, improving housing conditions, and expanding primary healthcare infrastructure in marginalized communities are areas that could directly reduce transmission risks. Furthermore, regular deworming campaigns, with catching stray dogs and popularisation of this issue, targeting vulnerable groups, may further lower the burden of infection. In addition, GIS-based risk mapping can help authorities distribute resources more efficiently, ensuring that interventions reach the most affected populations. Together, these insights may provide valuable information to reduce parasitic disease burdens and improve health equity in other marginalized or hard-to-reach populations across different geopolitical settings.

### 4.6. Limitations

This study faced several limitations. One was the low number of samples in some areas. In some municipalities, the sample size was very small, which may have slightly distorted the results, particularly in the positivity maps, by underrepresenting local prevalence. Another challenge was that the study also relied on one-time sampling, which, while logistically simpler, limits data reliability. Temporal heterogeneity across the study period may have influenced the results and should also be considered a limitation. More frequent, longer-term and repeated sampling in the same areas could improve data accuracy and allow observation of seasonal differences. However, such an approach poses ethical challenges and risks undermining trust, particularly in marginalised communities, and was therefore not applied in this study. Also, we were using older available data, which was less problematic for municipalities than for Roma settlements, where high mobility and seasonal changes can change living conditions and population composition a few times in year. Addressing these problems requires careful planning and community engagement, which can be time-consuming due to bureaucratic and ethical processes. Finally, the study used only microscopic concentration methods without molecular tools like PCR. Although microscopy is cost-effective and suitable for detecting helminths, it likely underestimates protozoan prevalence. PCR was not feasible due to sample size and resource limits, still our methods detected infections such as *Giardia* in some cases.

## 5. Conclusions

This study highlights and underlines the importance of preventive screening of the epidemiological situation in selected areas in Slovakia. As we could see from the results, intestinal endoparasites remain present in the Slovak population, with considerably higher prevalence in certain areas. We detected more positive samples, mainly from the Roma population, people living in rural areas and children. In addition, identifying specific risk areas and understanding the associated risk factors are essential for effective disease prevention. This knowledge enables authorities, municipalities, and other stakeholders to direct funding, resources, and interventions more precisely, thus focusing efforts on communities and areas most at risk. These findings suggest that the main preventive measure for lowering the risk of endoparasite transmission should be to improve the socio-economic and hygienic conditions of the population or to comply with the basic principles of personal hygiene. At the same time, ensuring access to safe drinking water and adequate sanitation facilities is essential, especially in localities with a predominance of marginalised populations, to avoid further contamination of the environment with endoparasitic stages. In addition, veterinary care for domestic animals needs to be improved with long-term monitoring of the effectiveness of these measures. Finally, it is important to stress that spatial analyses are of great importance in predicting disease outbreaks, as was demonstrated during the recent COVID-19 epidemic. Thus, the integration of epidemiological and spatial information will allow for a more accurate and efficient public health response in dealing with infectious (including parasitic) diseases, thereby also increasing the effectiveness of preventive measures and targeted interventions.

## Figures and Tables

**Table 1:** Number of positive human stool samples divided according to parasite species ethnicity and sex.

Eggs/Cysts	Overall	Ethnicity				Sex			
		Roma		Non-Roma		Men		Women	
		*n* *	%	*n* *	%	*n* *	%	*n* *	%
***A. lumbricoides***	159	146	91.82	13	8.18	82	51.57	77	48.43
***T. trichiura***	20	18	90.00	2	10.00	14	70.00	6	30.00
***E. vermicularis***	14	13	92.86	1	7.14	10	71.42	4	28.57

*n*—number of samples detected with eggs of given parasite; %—percentage of number of samples detected with eggs of given parasite; *—this includes just the number of positive samples with the given intestinal parasites detected; it does not refer to the number of people infected.

**Table 2:** Key epidemiological metrics calculated from our parasitological data, categorised by overall sample size, ethnicity, sex, and environment.

		*n*	*n*_posit_	% (95% CI)	COR (95% CI)	χ^2^	*p*-Value
**Overall**		3816	193	5.06 (4.38–5.80)	-	-	-
**Nationality**	**Roma**	1302	176	13.52 (11.70–15.49)	22.95 (13.88–37.95)	294.56	<0.01 *
	**Non-Roma ^a^**	2514	17	0.68 (0.39–1.08)	0.04 (0.02–0.07)	-	-
**Sex**	**Men**	1972	102	5.17 (4.24–6.25)	1.05 (0.78–1.40)	0.11	0.74
	**Women ^a^**	1844	91	4.93 (3.99–6.02)	0.95 (0.71–1.27)	-	-
**Environment**	**Rural**	1819	124	6.82 (5.70–8.07)	2.04 (1.51–2.76)	22.40	<0.01 *
	**Urban ^a^**	1997	69	3.46 (2.69–4.35)	0.48 (0.36–0.66)	-	-

*—*p* < 0,01; ^a^—reference group for calculating OR in other group; *n*—number of samples; *n*_posit_—number of positive samples; %—percentage of positive samples; 95% CI—95% Confidence interval; COR—Crude odds ratio; χ^2^—chi squared test.

**Table 3:** Key epidemiological metrics calculated from our parasitological data, categorised by age groups.

		*n*	*n*_posit_	% (95% CI)
**Age groups**	**Infants (0–1)**	220	14	6.36 (3.52–10.44)
	**Kids (2–6)**	1128	70	6.21 (4.86–7.77)
	**Adolescents (7–18)**	1186	85	7.17 (5.76–8.78)
	**Productive (19–66)**	1146	23	2.01 (1.27–2.99)
	**Post-productive (>66)**	136	1	0.74 (0.00–4.02)

*n*—number of samples; *n*_posit_—number of positive samples; %—percentage of positive samples; 95% CI—95% Confidence interval.

## Data Availability

The dataset used in this publication is not publicly available due to safety concerns for our participants, but it is available from the first author and the corresponding author upon reasonable request.
